# Acquired Factor XI Deficiency during SARS-CoV-2 Infection: Not Only Thrombosis

**DOI:** 10.1055/s-0040-1714696

**Published:** 2020-09-13

**Authors:** Giacomo Andreani, Lorenzo Uscello, Barbara Montaruli, Antonio Briozzo, Francesco Vitale, Marinella Tricarico, Luisa Arnaldi, Stefania Marengo, Claudio Norbiato

**Affiliations:** 1 Department of Clinical and Biological Sciences, University of Turin, Turin, Italy; 2 Department of Internal Medicine, A.O. Ordine Mauriziano, Turin, Italy; 3 Laboratory Analysis, A.O. Ordine Mauriziano, Turin, Italy

An 80-year-old woman with a positive RT-PCR (reverse transcription polymerase chain reaction) for severe acute respiratory syndrome coronavirus 2 (SARS-CoV-2) on nasopharyngeal swab was admitted to our COVID-19 department during the pandemic with fever, dyspnea, and need for oxygen therapy; two large hematomas were present bilaterally in the axillary zones. Her past medical history included Crohn's disease diagnosed 30 years ago after perforation of the ascending colon, hypertension, bilateral hip replacement, appendectomy, and tonsillectomy; no history of bleeding complications was reported during or after surgical interventions or in the postpartum, and no history of menorrhagia or easy-bruising. One month and a half before admission, she underwent right knee replacement surgery with no complications, no blood units, or plasma transfusions were required. At that time complete blood count and screening clotting test were within normal range.

Investigations on admission were as follows: white blood cell 11,380/mm ^3^ (Neu 10,790; Ly 240); hemoglobin 8 g/dL; mean corpuscular volume 89 fL; platelets 251,000/mm ^3^ ; PTr 1.16 (n.v. 0.80–1.21); PTTr 1.49 (n.v. 0.80–1.18); D-dimer 2,161 μg/L (n.v. < 790); fibrinogen 455 mg/dL (n.v. 180–400); C-reactive protein 78.4 mg/L (n.v. < 5.0). A chest computed tomography scan outlined a picture of bilateral interstitial pneumonia with ground-glass areas and thickening of interstitium, consistent with a viral infection; moreover, the presence of hyperdense hematomas in the axillary zones was recorded (thickness 20–30 mm), no active bleeding was detected. Antiretroviral therapy with darunavir and ritonavir was started; hydroxychloroquine was not administered for prolonged QTc on electrocardiogram. Enoxaparin 4000 IU/day was introduced for prophylaxis and continued until hospital discharge.

The isolated prolonged PTTr (silica) was confirmed in day +5 and +7 from admission (1.30 and 1.42, respectively), PTTr (ellagic acid, reagent sensible to factors VIII, IX, XI deficiency) was prolonged too, so a PTT MIX test was required: the PTTr MIX at time 0 was normal but PTTr MIX at 2 hours at 37°C was above the normal range, consistent with the presence of an inhibitor ( Table 1 ); the fXI activity was 37% (n.v. 55–150) while fVIII, fIX, and fXII were in the normal range. Detection of antiphospholipid antibodies (lupus anticoagulant, anticardiolipin, and anti-β2 glycoprotein I) resulted negative. The PTT MIX test and clotting factors activity was retested 3 days later and the presence of an acquired deficiency of fXI was confirmed. Patient was discharged in good clinical conditions more than 1 month after admission, no thrombotic or bleeding complications occurred during hospitalization.

**Table 1:** Coagulatory and inflammatory parameters

	Unit	Ref.	March 17	March 21	March 23	March 26	April 7	April 12	April 18	April 21
Thrombin time	Ratio	[< 1.20]			1.08					
aPTTr (silica)	Ratio	[0.80–1.18]	1.49	1.30	1.42	1.55	1.40	1.30	1.30	1.24
aPTT MIX time 0	Ratio	[0.80–1.18]			1.17	1.24				
aPTT MIX 2 h 37°C	Ratio	[0.80–1.18]			1.20					
aPTTr (ellagic acid)	Ratio	[0.70–1.18]			1.36					
Factor VIII	%	[50–200]			120					
Factor IX	%	[65–140]			101					
**Factor XI**	**%**	**[55–150]**			**37**	**38.5**				
Factor XII	%	[55–180]			63					
D-dimer	μg/L	[< 790]	2,161		3,501		2,471	2,004	2,183	
Fibrinogen	mg/dL	[180–400]	455	541	505			541	568	
CRP	mg/L	[< 5.0]	78.4			55.6		12.7	34.7	

Abbreviations: aPTTr, activated partial thromboplastin time ratio; CRP, C-reactive protein.

Inhibitors against clotting factors generally occur in patients with severe congenital deficiencies who underwent periods of replacement therapy. Acquired factor inhibitors in the absence of congenital deficiency are rare events, among them the production of autoantibodies inhibiting fVIII is the most frequent acquired bleeding disorder. On the contrary, inhibitors against fXI have been reported only anecdotally in literature mostly associated to autoimmune disorders, that is, systemic lupus erythematosus, 1 rheumatoid arthritis, 2 Crohn's disease, 3 membranoproliferative glomerulonephritis, 4 or in association with malignancies, that is, chronic lymphocytic leukemia, 5 gastrointestinal adenocarcinoma, 3 and thymoma 6 ; a case of transient acquired fXI deficiency after gynecological surgery has been reported. 7 The risk of bleeding in conditions of fXI deficiency is relatively low and correlation between factor levels and symptoms is very poor; in these persons hemorrhage is usually provoked, exacerbated by trauma or surgical procedures. 8 9

In the classical chart of the *intrinsic pathway* of coagulation waterfall the fXIIa activates the zymogen fXI which in turn activates fIX and so, through a series of enzymatic reactions, it conducts to thrombin formation. It is nowadays well documented that fXII has only a marginal role in the hemostasis in vivo, contributing more to inflammation and pathologic coagulation; in fact, fXI seems to be activated in a stronger way directly by thrombin, thus supporting and amplifying thrombin formation in loop. 10 Moreover, it has also been shown how fXIa activates fXII after tissue factor-initiated coagulation (via *extrinsic pathway* ). 11 Maybe coagulation is not a waterfall but a whirlpool!

But fXI is not only a component of the coagulation pathway, it also participates to the process of contact activation by mean of the bradykinin-generating kallikrein–kinin system (KKS) (including fXII, prekallikrein PK, high molecular weight-kininogen HK, and C1 esterase inhibitor C1INH) contributing in this way to several host-defenses and to the initial response to infection: in fact, the inflammatory response to viral infections is in part mediated by contact activation and it has already been demonstrated how hantavirus and herpes simplex virus 1 can lead to the activation of KKS pathway. 12 The fXI, together with fXII, seems to have a pivotal role in this context. 13 In a sepsis setting of fXI deficient mice, Bane et al have shown how a reduced early cytokine response and KKS activation conduct to a survival benefit compared with wild-type mice. 11 14

Reconnecting to the case reported above, the reason for the occurrence of inhibitors production against fXI during SARS-CoV-2 infection is still not clear to us, but maybe we can speculate that the inhibition of fXI could be a means of the immune system to try to turn off a sustained inflammatory overresponse against an infective agent which can otherwise conduct to deleterious effects for the host. Why not to consider this patient, with an autoimmune *milieu* (Crohn's disease), as evolutionary advantaged in this condition having the possibility to counterbalance the state of hypercoagulability and inflammation (the so-called, thromboinflammation 15 )? Why not to act directly against fXI, inhibiting it and on one side blocking the pathological coagulation loop with a mild risk of hemorrhage, as already demonstrated 16 17 and on the other side disrupting the mechanism driving to the uncontrolled inflammatory overresponse by blunting KKS and complement activation?

By our, at this point, everyday experience and from the still scanty literature in this regard it seems to us that patients with severe pneumonias by SARS-CoV-2 can develop a hypercoagulable condition driving to a higher risk for (micro)vascular thrombosis, still to demonstrate 18 19 20 21 ; if true, considering the interface role of fXI in vascular thrombosis and inflammation, the development of therapies inhibiting this factor could be an intriguing leverage on which to act for trying to improve microvascular perfusion, reduce inflammation, and protect organ function during severe infections.

## References

[1] Wool G D Treml A Miller J L Acquired factor XI deficiency and therapeutic plasma exchange J Clin Apher 2018 33 03 427 430 29027258 10.1002/jca.21593

[2] Reece E A Clyne L P Romero R Hobbins J C Spontaneous factor XI inhibitors. Seven additional cases and a review of the literature Arch Intern Med 1984 144 03 525 529 6703824 10.1001/archinte.144.3.525

[3] Kyriakou D S Alexandrakis M G Passam F H Acquired inhibitors to coagulation factors in patients with gastrointestinal diseases Eur J Gastroenterol Hepatol 2002 14 12 1383 1387 12468962 10.1097/00042737-200212000-00016

[4] McManus M P Frantz C Gailani D Acquired factor XI deficiency in a child with membranoproliferative glomerulonephritis Pediatr Blood Cancer 2012 59 01 173 175 21850674 10.1002/pbc.23287 PMC4364028

[5] Goodrick M J Prentice A G Copplestone J A Pamphilon D H Boon R J Acquired factor XI inhibitor in chronic lymphocytic leukaemia J Clin Pathol 1992 45 04 352 353 1577975 10.1136/jcp.45.4.352 PMC495279

[6] Jethava Y Alamelu J Rangarajan S Lang-Lazdunski L van der Walt J Fields P Acquired agranulocytosis and factor XI deficiency in association with thymoma J Clin Oncol 2011 29 20 e604 e606 21537052 10.1200/JCO.2010.34.3707

[7] Castaman G Ruggeri M Rodeghiero F Acquired transitory factor XI inhibitor after gynaecological surgery Haemophilia 2008 14 03 643 644 18312367 10.1111/j.1365-2516.2008.01677.x

[8] Wheeler A P Gailani D Why factor XI deficiency is a clinical concern Expert Rev Hematol 2016 9 07 629 637 27216469 10.1080/17474086.2016.1191944 PMC4950943

[9] Peyvandi F Palla R Menegatti M Coagulation factor activity and clinical bleeding severity in rare bleeding disorders: results from the European Network of Rare Bleeding Disorders J Thromb Haemost 2012 10 04 615 621 22321862 10.1111/j.1538-7836.2012.04653.x

[10] Emsley J McEwan P A Gailani D Structure and function of factor XI Blood 2010 115 13 2569 2577 20110423 10.1182/blood-2009-09-199182 PMC4828079

[11] Bane C E Jr Ivanov I Matafonov A Factor XI deficiency alters the cytokine response and activation of contact proteases during polymicrobial sepsis in mice PLoS One 2016 11 04 e0152968 27046148 10.1371/journal.pone.0152968 PMC4821616

[12] Long A T Kenne E Jung R Fuchs T A Renné T Contact system revisited: an interface between inflammation, coagulation, and innate immunity J Thromb Haemost 2016 14 03 427 437 26707513 10.1111/jth.13235

[13] Shatzel J J DeLoughery E P Lorentz C U The contact activation system as a potential therapeutic target in patients with COVID-19 Res Pract Thromb Haemost 2020 4 04 500 505 32542210 10.1002/rth2.12349 PMC7264624

[14] Tucker E I Verbout N G Leung P Y Inhibition of factor XI activation attenuates inflammation and coagulopathy while improving the survival of mouse polymicrobial sepsis Blood 2012 119 20 4762 4768 22442348 10.1182/blood-2011-10-386185 PMC3367876

[15] Jackson S P Darbousset R Schoenwaelder S M Thromboinflammation: challenges of therapeutically targeting coagulation and other host defense mechanisms Blood 2019 133 09 906 918 30642917 10.1182/blood-2018-11-882993

[16] Chen W Carvalho L P Chan M Y Kini R M Kang T S Fasxiator, a novel factor XIa inhibitor from snake venom, and its site-specific mutagenesis to improve potency and selectivity J Thromb Haemost 2015 13 02 248 261 25418421 10.1111/jth.12797

[17] Büller H R Bethune C Bhanot S Factor XI antisense oligonucleotide for prevention of venous thrombosis N Engl J Med 2015 372 03 232 240 25482425 10.1056/NEJMoa1405760 PMC4367537

[18] Magro C Mulvey J J Berlin D Complement associated microvascular injury and thrombosis in the pathogenesis of severe COVID-19 infection: a report of five cases Transl Res 2020 220 1 13 32299776 10.1016/j.trsl.2020.04.007 PMC7158248

[19] Terpos E Ntanasis-Stathopoulos I Elalamy I Hematological findings and complications of COVID-19 Am J Hematol 2020 95 07 834 847 32282949 10.1002/ajh.25829 PMC7262337

[20] Klok F A Kruip M JHA van der Meer N JM Incidence of thrombotic complications in critically ill ICU patients with COVID-19Thromb Res2020 10.1016/j.thromres.2020.04.013 PMC7146714 32291094

[21] Panigada M Bottino N Tagliabue P Hypercoagulability of COVID-19 patients in intensive care unit: a report of thromboelastography findings and other parameters of hemostasis J Thromb Haemost 2020 10.1111/jth.14850 PMC9906150 32302438

